# Bioactive lipid lysophosphatidic acid species are associated with disease progression in idiopathic pulmonary fibrosis

**DOI:** 10.1016/j.jlr.2023.100375

**Published:** 2023-04-17

**Authors:** Margaret Neighbors, Qingling Li, Sha (Joe) Zhu, Jia Liu, Weng Ruh Wong, Guiquan Jia, Wendy Sandoval, Gaik W. Tew

**Affiliations:** 1 OMNI Translational Medicine, Genentech Inc., South San Francisco, USA; 2 Department of Microchemistry, Proteomics & Lipidomics, Genentech Inc., South San Francisco, USA; 3 PD Data Science, F Hoffmann-La Roche, Shanghai, China; 4 Department of Biomarker Discovery OMNI, Genentech Inc., South San Francisco, USA; 5 I2O Technology and Translational Research, Genentech Inc., South San Francisco, USA

**Keywords:** Lysophosphatidic acid, DLCO, exacerbation, mortality, idiopathic pulmonary fibrosis

## Abstract

Idiopathic pulmonary fibrosis (IPF) is a progressive disease with significant mortality. Prognostic biomarkers to identify rapid progressors are urgently needed to improve patient management. Since the lysophosphatidic acid (LPA) pathway has been implicated in lung fibrosis in preclinical models and identified as a potential therapeutic target, we aimed to investigate if bioactive lipid LPA species could be prognostic biomarkers that predict IPF disease progression. LPAs and lipidomics were measured in baseline placebo plasma of a randomized IPF-controlled trial. The association of lipids with disease progression indices were assessed using statistical models. Compared to healthy, IPF patients had significantly higher levels of five LPAs (LPA16:0, 16:1, 18:1, 18:2, 20:4) and reduced levels of two triglycerides species (TAG48:4-FA12:0, -FA18:2) (false discovery rate < 0.05, fold change > 2). Patients with higher levels of LPAs had greater declines in diffusion capacity of carbon monoxide over 52 weeks (*P* < 0.01); additionally, LPA20:4-high (≥median) patients had earlier time to exacerbation compared to LPA20:4-low (<median) patients (hazard ratio (95% CI)): 5.71 (1.17–27.72) (*P* = 0.031). Higher baseline LPAs were associated with greater increases in fibrosis in lower lungs as quantified by high-resolution computed tomography at week 72 (*P* < 0.05). Some of these LPAs were positively associated with biomarkers of profibrotic macrophages (CCL17, CCL18, OPN, and YKL40) and lung epithelial damage (SPD and sRAGE) (*P* < 0.05). In summary, our study established the association of LPAs with IPF disease progression, further supporting the role of the LPA pathway in IPF pathobiology.

Idiopathic pulmonary fibrosis (IPF) is a heterogeneous disease of unknown etiology and high mortality with a median survival of about 3 years from the time of disease diagnosis (1, 2). The rate of disease progression is highly variable among patients, but acute declines in lung function and respiratory failure occur in approximately 10% of patients every year (3). Acute exacerbations (AE-IPF) are the most common cause of death among patients with IPF; their onsets are unpredictable and can progress rapidly with a 50% in-hospital mortality rate (4). Biomarkers that could predict disease progression and AE-IPF are urgently needed to better manage the disease and improve the clinical trial design.

The autotaxin-lysophosphatidic acid (ATX-LPA) signaling pathway has been implicated in lung fibrosis in preclinical models (5, 6). ATX generates the majority of the bioactive lipid LPA detected in blood and inflamed tissues (7, 8). LPA signals through G protein–coupled LPA receptors (LPAR_1-6_) expressed on many tissues and immune cells (9) to mediate lymphocyte homing (6, 7) and promote fibrosis and vascular leakage (10). LPA levels were increased in bronchoalveolar lavage fluid following lung injury in a mouse bleomycin model of pulmonary fibrosis, and the deletion of one of its receptors, LPA_1_, protected mice from fibrosis and mortality (10). Normal human bronchial epithelial cells treated with LPA caused stress fiber formation and integrin αvβ6 re-organization leading to transforming growth factor beta (TGF-β) activation, linking LPA to TGF-β responses, and establishing LPA as an important profibrotic factor (11).

LPA species vary in length and fatty acid saturation. In patients with IPF, LPA22:4 levels were elevated in the exhaled breath condensate compared to the control (12). Lysophosphatidylcholine (LPC), a precursor of LPA, was also found to be higher in patients with IPF compared to controls in an independent study (13). Consistently, a number of LPA precursors, as well as triglyceride (TAG) species, were shown to be upregulated in patients with IPF having a progressive disease as compared to patients with stable disease (14). In a phase 2a study in patients with IPF, ATX inhibitor reduced plasma LPA18:2 levels by at least 50% over 12 weeks, and the reduction was accompanied by forced vital capacity (FVC) stabilization in the treated group while the placebo group showed a trend of FVC decline (15). Together, this evidence supports further study of the potential of LPA and lipids as disease biomarkers in IPF.

As different LPA and lipid species have been reported in IPF and other respiratory diseases, we performed targeted assays that measured LPA species (LPA16:0, 16:1, 18:0, 18:1, 18:2, 20:4, and 22:4) as well as global lipidomic profiling to identify specific LPA and lipid species that were dysregulated in patients with IPF and assessed the relationship between the dysregulated lipids and disease progression.

## Materials and methods

### Study cohort

Available baseline plasma samples from the placebo arm of the IPF randomized control trial CAPACITY-006 (NCT00287729) were used for LPA (n = 102) and global lipidomic (n = 99) measurements. The design of the clinical trial has been described (16). Briefly, patients aged 40–80 years with a diagnosis of IPF in the previous 48 months, with FVC%pred of 50–90%, percentage of predicted diffusion capacity of carbon monoxide (DLCO%pred) of 35–90%, and 6-min walk distance of at least 150 m were enrolled and observed for 72 weeks. High-resolution computed tomography images (HRCT) captured at screening and week 72 were analyzed by classifier-based quantitative image analysis scoring. Age- and sex-matched healthy controls (n = 30) from an internal biobank were used for comparison.

The study was approved by independent ethics committees and institutional review boards at each institution and by InterMune Inc or designees and conducted according to the standards of the Declaration of Helsinki. Written informed consent was obtained from participants prior to biomarker analysis.

### Mass spectrometry LPA assay

LPA species were measured using a targeted method (17). Briefly, 500 μl disodium phosphate buffer and 2 ml butanol were added to 20 μl plasma to extract lipids. The extracted samples were reconstituted in methanol and analyzed by liquid chromatography-mass spectrometry (LC-MS/MS), with LC coupling to a quadrupole linear ion trap (QTRAP) mass spectrometer employed under negative ionization mode. High-performance liquid chromatography separation of LPA was optimized on a C18 column to separate LPA from other lipids. Sample analysis was performed in multiple reactions monitoring mode. LPA species were identified and quantified based on characteristic mass spectrometry transitions, retention time, and an internal standard (C13-LPA16:0). Additional LPA species (LPA16:0, 18:0, 18:1, 18:2, 20:4) standards (Avanti Polar Lipids, Alabaster, AL) and the internal standard were used to generate absolute concentration standard curve, and the remaining LPA species (LPA16:1, 22:4) were reported as ratio-to-internal-standard (rts) as the measurement unit. To avoid interference from in-source fragmentation, LPAs were separated from other lysophospholipids on the column.

### Lipidomic profiling

Patients with sufficient remaining plasma volume (n = 99) were used for lipidomic profiling. Lipidomic measurement was performed with a modified method derived from a previous study (18) using the Lipidyzer™ platform (19). Briefly, 54 isotope-labeled internal standards were added into the sample before lipid extraction. Lipids were purified using dichloromethane, methanol, and water in two phases extraction. Lipid species were analyzed on a SelexION-enabled 6,500 QTRAP mass spectrometry (Sciex, Redwood City, CA) in multiple reaction monitoring mode by direct infusion. Lipid species were identified and quantified based on characteristic MS transitions and internal standards.

### Blood protein biomarker assays

Rationales and details of the protein biomarker measurements have previously been described (20). Briefly, plasma biomarkers cartilage oligomeric matrix protein (COMP), periostin (POSTN), CC motif chemokine ligand 17 (CCL17), CC motif chemokine ligand 18 (CCL18), and interleukin 13 (IL13) were measured using the IMPACT platform. ELISA was used to measure CXC chemokine ligand 13 (CXCL13; R&D Systems, Minneapolis, MN, USA), Luminex for matrix metalloproteinase 7 (MMP7; eBioscience, San Diego, CA, USA), prototype Elecsys platforms (Roche Diagnostics, Penzberg, Germany) for CXC chemokine ligand 14 (CXCL14), osteopontin (OPN) and chitinase-3-like protein 1 (YKL40), and ProteinSimple platform (Biotechne, Minneapolis, MN, USA) for surfactant protein-D (SPD) and soluble receptor for advanced glycation end products (sRAGE).

### Statistical analysis

Statistical analyses were performed using R (version 3.6.3). LPA, lipid, and protein biomarker concentrations were log2 transformed when appropriate. LPA and lipid concentrations were compared between healthy controls and IPF patients, using multivariate regression adjusting for age and sex, followed by Benjamini-Hochberg correction (false discovery rate [FDR]) for multiple comparisons. FDR <0.05 was considered statistically significant. The relationship between LPA and lipid levels with baseline demographics and protein biomarkers was assessed using Student’s *t* test, Spearman correlation, or univariate and multivariate linear regression adjusting for age, sex, and geographical regions (United States vs. rest of the world).

FVC %pred or DLCO %pred slope was calculated using linear regression in patients with at least three measurements over 52 weeks. The absolute differences in HRCT indices between screening and week 72 were calculated. Since some of the LPA levels were significantly different between female and male patients, sex-specific median levels of each LPA and TAG species were used to dichotomize patients into biomarker high (≥ median) and low (< median) subgroups. The median cutoff concentrations or ratio-to-standards are shown in the supplemental Table S1. All models used to estimate the association of biomarkers with disease progression included age, sex, geographical region, baseline FVC %pred, and DLCO %pred as covariates. Multivariate linear and logistic regression models were used to assess the association of biomarkers with continuous clinical outcomes (decline in FVC %pred, DLCO %pred, and changes in HRCT indices), and death, respectively. Cox proportional hazards regression model was used to compare the time to first exacerbation or respiratory hospitalization. The statistically significant biomarkers were subsequently included in incremental models to identify independent prognostic biomarkers while adjusting for the aforementioned covariates. *P* value < 0.05 in multivariate models was considered statistically significant. Reported prognostic protein biomarkers were included in the analyses as benchmarks for the lipid biomarkers.

## Results

### Differences in lipids between IPF and healthy controls

The baseline characteristics of patients with IPF and the age- and sex-matched healthy controls are shown in Table 1. The samples used in this study were representative of the overall placebo patients of the trial as there were no significant differences in patient demographics or lung function measures between the cohorts.

**Table 1:** Patient baseline characteristics of CAPACITY-006 study

Characteristics	CAPACITY-006				Healthy
	All Placebo	LPA	Global lipid	*P*	
	N = 173	N = 102	N = 99	All versus LPA,	N = 30
				All versus Global	
Age (years)	67 (7·8)	67 (8·0)	66·7 (8·0)	0·99, 0·78	67 (6·0)
Male %	124 (71·7%)	74 (72·5%)	71 (71·7%)	0·99, 1·0	24 (80%)
Race					
White, %	171 (98·8%)	102 (100%)	99 (100%)		27 (90%)
Black or African American, %	2 (1·2%)	0	0	0·72, 0·74	1 (3·3%)
Others, %	0	0	0		2 (6·7%)
Ever smokers, %	109 (63·0%)	64 (62·7%)	62 (62·6%)	0·79, 0·83	
FVC % predicted	73·1 (14·2)	72·8 (14·1)	73 (14·2)	0·86, 0·94	
DLCO % predicted	47·4 (9·2)	47·9 (9·6)	48·1 (9·6)	0·77, 0·59	
Six-minute walk distance (meter)	399·1 (89·7)	406.4 (95·9)	407·1 (96·5)	0·54, 0·52	

DLCO%pred, percentage of predicted diffusion capacity of carbon monoxide; FVC%pred, percentage of predicted forced vital capacity.

Data are n (%), mean (SD).

**Fig. 1:**
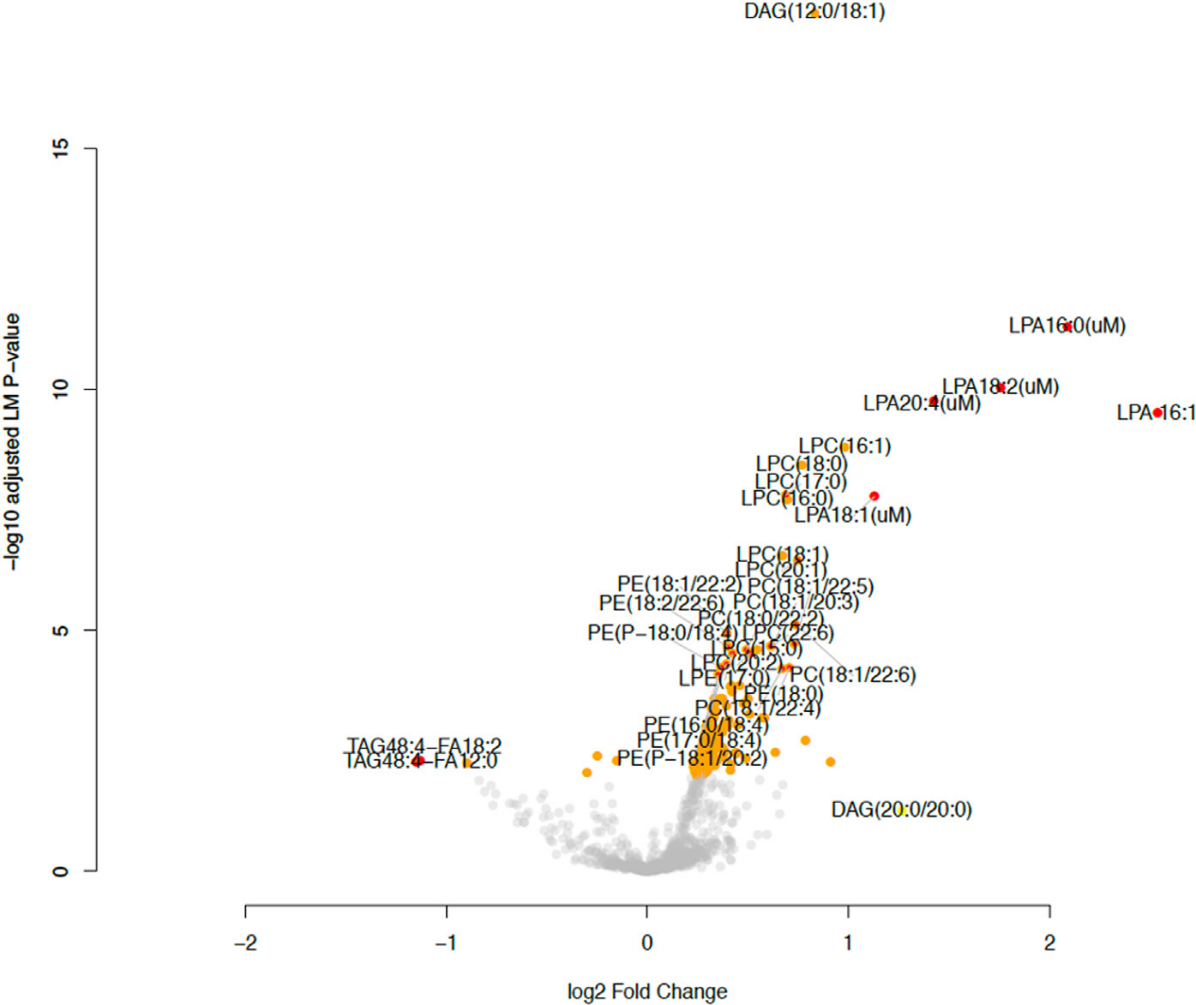
Comparison of lipid levels between healthy controls and IPF. A multivariate linear regression model adjusted for age and sex was used to assess the differences in lipid levels between healthy controls and patients with IPF. The x-axis indicated log2(analyte abundance in IPF/analyte abundance in healthy controls), and the y-axis indicates −log10(adjusted *P*-value or false discovery rate of the multivariate regression). Yellow circles denote lipid species with false discovery rate <0.05; red circles denote lipid species with false discovery rate <0.05 and fold change >2.

A total of 206 lipid species were significantly upregulated in patients with IPF, including many of the LPA precursors such as phosphatidylcholine (PC), phosphatidylethanolamine (PE), LPC, and lysophophatidylethanolamine species; a total of 22 lipid species were significantly downregulated in patients with IPF compared to controls (FDR < 0.05) (supplemental Table S2). Among these lipids, only seven species had a fold change greater than two: LPA16:0, 16:1, 18:1, 18:2, 20:4 and TAG species TAG48:4: fatty acid (FA)12:0 and TAG48:4-FA18:2 (Fig. 1).

We focused on these 7 lipid species and assessed their baseline association with demographic and clinical measures. In healthy controls, LPA18:2 levels were higher in females than males (*P* < 0.05) (supplemental Fig. S1A). In patients with IPF, only LPA16:1 levels were higher in females compared to males (*P* < 0.05) (supplemental Fig. S1B), while LPA20:4 showed a modest negative association with 6-min walk distance (*P* = 0.0884) (supplemental Fig. S1C). No other association with respect to other baseline characteristics (age, FVC, DLCO) was observed for these seven lipid species (not shown).

### Association with profibrotic and lung epithelial damage markers

**Fig. 2:**
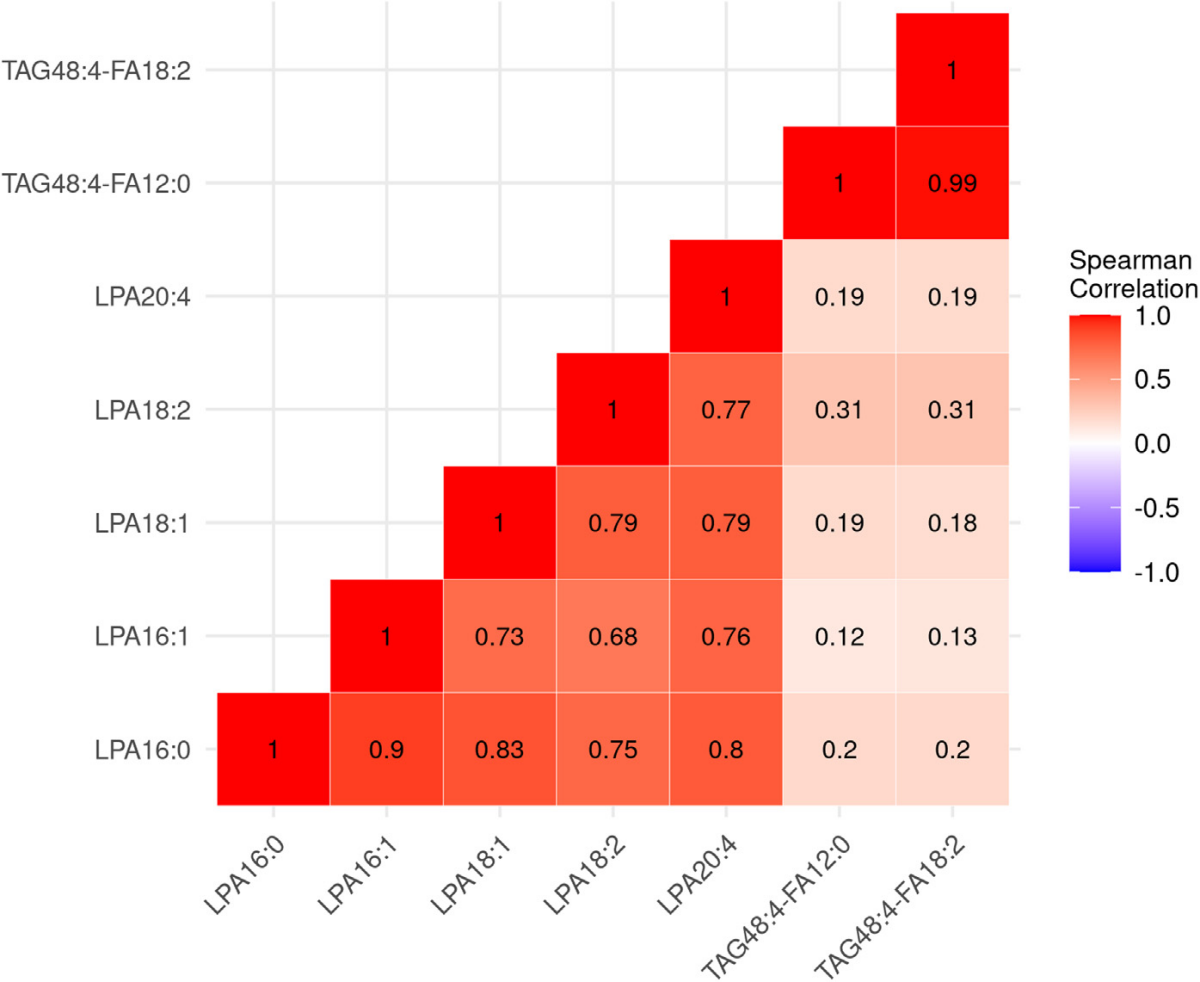
Baseline correlation of lipid species. Spearman’s rho value shown.

In IPF patients, LPA species were intercorrelated (rho 0.68–0.90) but showed little to no correlation with the two TAG species (rho 0.12–0.31) (Fig. 2). The TAG species were highly correlated with each other (rho = 0.99) (Fig. 2). A number of LPA species were positively associated with reported prognostic biomarkers such as CCL17, CCL18, COMP, OPN, and YKL40, as well as lung epithelial damage biomarkers such as SPD and sRAGE (*P* < 0.05); TAG species had a significant negative association with CXCL13 (*P* < 0.05) (Table 2). The correlation of the protein biomarkers has been described (20) except for SPD and sRAGE which were now included (supplemental Fig. S2).

**Table 2:** Baseline correlation of lipids and protein biomarkers

Lipid species	CXCL13			CXCL14			CCL17			CCL18		
	Coefficient	SE	*P*	Coefficient	SE	*P*	Coefficient	SE	*P*	Coefficient	SE	*P*
LPA16:0	0.035	0.089	0.694	−0.587	0.413	0.162	0.093	0.042	0.030∗	0.228	0.09	0.013∗
LPA16:1	0.016	0.098	0.868	−0.306	0.475	0.521	0.086	0.049	0.08	0.244	0.105	0.022∗
LPA18:1	−0.016	0.078	0.834	−0.701	0.324	0.035∗	0.081	0.036	0.026∗	0.129	0.08	0.112
LPA18:2	−0.041	0.089	0.643	−0.416	0.371	0.268	0.027	0.042	0.52	0.112	0.093	0.233
LPA20:4	0.002	0.081	0.978	−0.224	0.364	0.54	0.104	0.038	0.007∗	0.159	0.084	0.06
TG48:4-FA12:0	−0.34	0.154	0.030∗	0.254	0.59	0.669	−0.022	0.073	0.763	−0.291	0.161	0.073
TG48:4-FA18:2	−0.358	0.146	0.016∗	0.301	0.562	0.594	−0.014	0.07	0.847	−0.277	0.153	0.074

	COMP			IL13			MMP7			OPN		
	Coefficient	SE	*P*	Coefficient	SE	*P*	Coefficient	SE	*P*	Coefficient	SE	*P*
LPA16:0	0.161	0.123	0.195	0.032	0.076	0.679	−0.029	0.052	0.576	0.169	0.138	0.224
LPA16:1	0.28	0.138	0.045∗	0.013	0.086	0.884	−0.014	0.06	0.822	0.166	0.156	0.293
LPA18:1	0.176	0.104	0.092	−0.021	0.062	0.738	−0.009	0.045	0.845	0.229	0.107	0.038∗
LPA18:2	0.223	0.118	0.062	0.02	0.073	0.782	−0.009	0.052	0.858	0.146	0.123	0.24
LPA20:4	0.149	0.11	0.179	−0.001	0.068	0.991	−0.014	0.048	0.769	0.079	0.12	0.514
TG48:4-FA12:0	−0.262	0.215	0.227	−0.02	0.124	0.87	−0.067	0.094	0.48	0.287	0.192	0.142
TG48:4-FA18:2	−0.251	0.204	0.222	−0.007	0.118	0.95	−0.066	0.09	0.466	0.266	0.183	0.154

	POSTN			YKL40			SPD			sRAGE		
	Coefficient	SE	*P*	Coefficient	SE	*P*	Coefficient	SE	*P*	Coefficient	SE	*P*
LPA16:0	0.262	0.152	0.088	0.108	0.094	0.255	0.104	0.073	0.16	0.315	0.129	0.016∗
LPA16:1	0.273	0.174	0.118	0.24	0.102	0.023∗	0.169	0.082	0.043∗	0.368	0.145	0.013∗
LPA18:1	0.216	0.129	0.098	0.13	0.074	0.086	0.089	0.063	0.162	0.234	0.113	0.041∗
LPA18:2	0.191	0.149	0.203	0.098	0.084	0.245	0.028	0.074	0.708	0.244	0.129	0.062
LPA20:4	0.153	0.139	0.272	0.108	0.081	0.189	0.125	0.066	0.063	0.312	0.117	0.009∗
TG48:4-FA12:0	−0.403	0.261	0.125	−0.144	0.146	0.328	−0.092	0.128	0.472	−0.352	0.242	0.15
TG48:4-FA18:2	−0.406	0.249	0.106	−0.122	0.14	0.387	−0.076	0.122	0.532	−0.376	0.229	0.104

SE, standard error.

Data derived from the multivariate linear regression adjusted for age, sex and geographic region.

∗*P* < 0.05 of multivariate regressions.

### Prognostic biomarkers of clinical outcomes

**Fig. 3:**
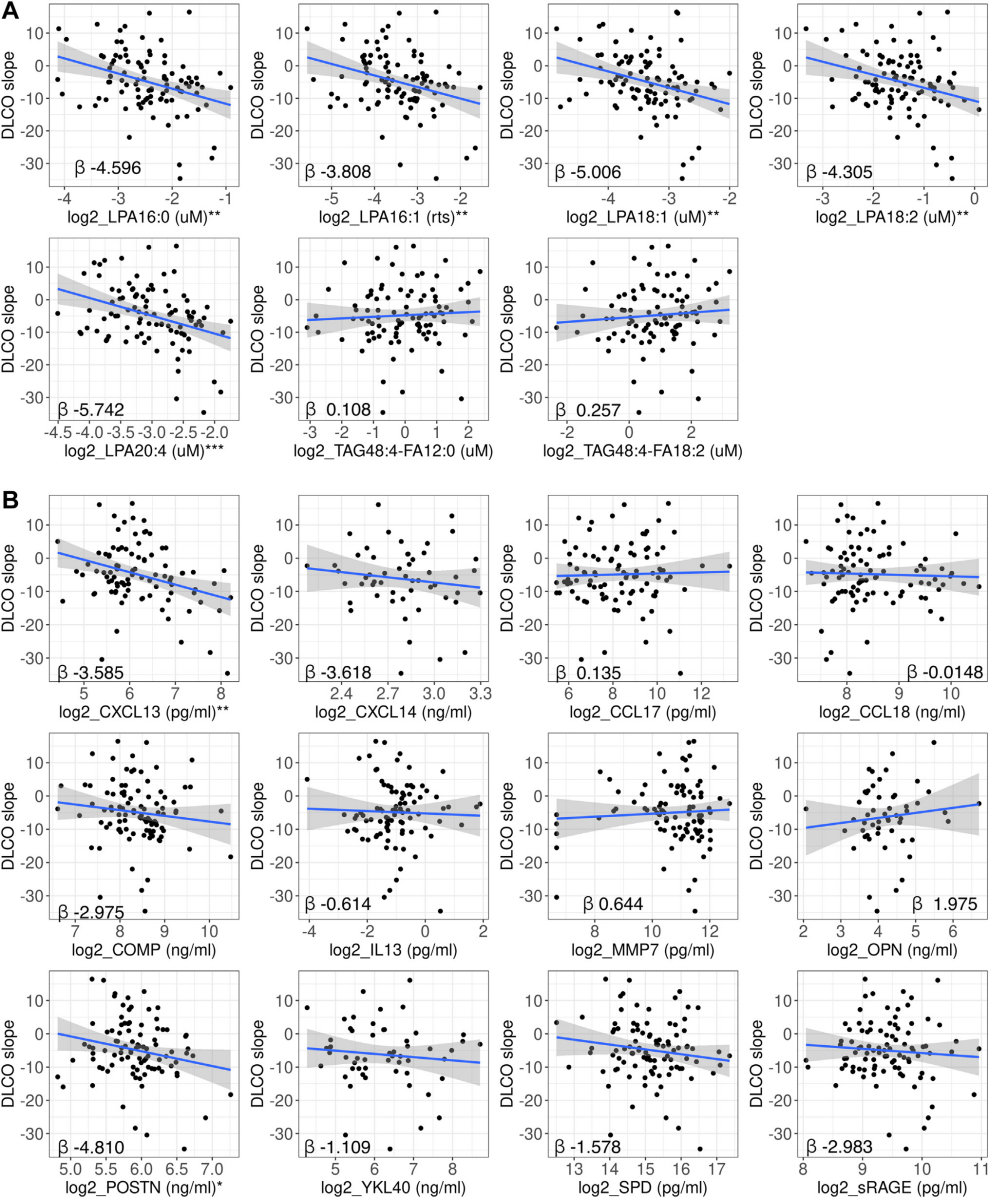
Decline in DLCO percentage predicted and baseline biomarker levels. A multivariate linear regression model adjusted for the following covariates: age, sex, baseline FVC %pred, baseline DLCO %pred, and the geographical region was used to assess the association between (A) LPA and TAG and (B) protein biomarkers, with DLCO %pred decline calculated as slope, where the y-axis indicated the DLCO %pred change per year. ∗∗*P* < 0.01; ∗∗∗*P* < 0.001 of multivariate linear regressions.

DLCO %pred slope of decline was significantly associated with all five of the LPA species (LPA16:0, 16:1, 18:1, 18:2, 20:4), where patients with higher levels of LPA at baseline had greater declines in DLCO %pred (*P* < 0.01–*P* < 0.001) (Fig. 3A). The absolute coefficients of the LPA models ranged from 3.81 to 5.74 (Fig. 3A), which showed that for every twofold increase in baseline LPA levels, the DLCO declined by an additional 3.81 %pred to 5.74 %pred per year, with all other covariables held constant. There was no association between TAG species and DLCO decline. Among the protein biomarkers, higher levels of CXCL13 and POSTN at baseline were associated with increased DLCO %pred decline (*P* < 0.05–*P* < 0.01) (Fig. 3B). To better understand the utility of the lipid biomarkers, combinations of the statistically significant lipid and protein biomarkers were included in incremental models to identify independent prognostic biomarkers. As all five of the LPA species were intercorrelated (Fig. 2), we did not include all of them in a model due to the issue of collinearity, the same applied to the TAG species. We first identified independent prognostic protein biomarkers and then combined the independent protein biomarker with the individual LPA or TAG species as predictors. For DLCO decline, CXCL13 remained a significant prognostic biomarker (*P* < 0.05) after adjusting for POSTN (Table 3). In the combined CXCL13 and individual LPA species model, CXCL13 and all the LPA species were independently associated with DLCO decline (*P* < 0.01–*P* < 0.001) (Table 3).

**Table 3:** Combination of prognostic biomarkers and clincial outcomes

DLCO Decline			
Combined protein biomarker model	Coefficient	SE	*P*
CXCL13 (log2 pg/ml)	−3.123	1.37	0.026∗
POSTN (log2 ng/ml)	−3.427	2.3	0.139

Combined protein & individual lipid biomarker model	Coefficient	SE	*P*
Combined CXCL13 and LPA16:0 model			
CXCL13 (log2 pg/ml)	−3.465	1.216	0.00549 ∗∗
LPA16:0 (log2 uM)	−5.649	1.372	8.85e-05 ∗∗∗
Combined CXCL13 and LPA16:1 model			
CXCL13 (log2 pg/ml)	−3.645	1.209	0.00337 ∗∗
LPA16:1 (log2 ratio-to-standard)	−5.204	1.22	5.14e-05 ∗∗∗
Combined CXCL13 and LPA18:1 model			
CXCL13 (log2 pg/ml)	−3.901	1.235	0.00219 ∗∗
LPA18:1 (log2 uM)	−5.897	1.561	0.000292 ∗∗∗
Combined CXCL13 and LPA18:2 model			
CXCL13 (log2 pg/ml)	−3.843	1.24	0.00264 ∗∗
LPA18:2 (log2 uM)	−4.904	1.346	0.000461 ∗∗∗
Combined CXCL13 and LPA20:4 model			
CXCL13 (log2 pg/ml)	−3.424	1.194	0.0052∗∗
LPA20:4 (log2 uM)	−6.71	1.469	1.66e-05 ∗∗∗

FVC decline			
Combined protein biomarker model	Coefficient	SE	*P*
CXCL13 (log2 pg/ml)	−2.441	1.404	0.0857
COMP (log2 ng/ml)	−5.868	1.772	0.00136∗∗

Exacerbation or respiratory hospitalization			
Combined protein & lipid biomarker model	Hazard ratio	95% CI	*P*
LPA20:4 (median)	3.050	0.42–22.17	0.271
YKL40 (median)	10.935	0.97–123.29	0.053

Mortality			
Combined protein biomarker model	Odd ratio	95% CI	*P*
CCL18 (median)	14.354	1.17–176.06	0.0372 ∗
SPD (median)	27.221	1.24–599.97	0.0363 ∗

Combined protein & individual lipid biomarker model 1			

CCL18 (median)	17.357	1.12–269.36	0.0414 ∗
SPD (median)	33.482	0.65–1724.27	0.0808
TAG48:4-FA12:0 (<median)	5.954	0.35–101.71	0.2181
Combined protein & individual lipid biomarker model 2			
CCL18 (median)	17.290	1.12–267.63	0.0414 ∗
SPD (median)	32.752	0.63–1706.82	0.0837
TAG48:4-FA18:2 (<median)	5.949	0.35–100.58	0.217

Fibrosis	Right lower lung			Left lower lung		
Combined protein & individual lipid biomarker model	Coefficient	SE	*P*	Coefficient	SE	*P*
Combined SPD and LPA16:1 model						
SPD (log2 pg/ml)	4.477	1.734	0.0124 ∗	4.819	1.679	0.00576 ∗∗
LPA16:1 (log2 ratio-to-standard)	5.895	2.004	0.00472 ∗∗	3.734	1.941	0.0594
Combined SPD and LPA16:0 model						
SPD (log2 pg/ml)	4.883	1.743	0.00694 ∗∗			
LPA16:0 (log2 uM)	5.453	2.111	0.0124 ∗			
Combined SPD and LPA18:1 model						
SPD (log2 pg/ml)	4.79	1.72	0.00727 ∗∗			
LPA18:1 (log2 uM)	6.836	2.349	0.00515 ∗∗			
Combined SPD and LPA18:2 model						
SPD (log2 pg/ml)	5.427	1.736	0.00279 ∗∗			
LPA18:2 (log2 uM)	5.465	2.19	0.0155 ∗			
Combined SPD and LPA20:4 model						
SPD (log2 pg/ml)	4.839	1.800	0.00939 ∗∗			
LPA20:4 (log2 uM)	4.783	2.508	0.0615			

CI, confidence interval; SE, standard error; uM, microMolar.

Values are from multivariate regression models predicting clinical outcomes of interest, using a combination of biomarkers as predictors while adjusting for the following covariates: age, sex, baseline FVC %pred, baseline DLCO %pred, and geographical region.

∗*P* < 0.05; ∗∗*P* < 0.01; ∗∗∗*P* < 0.001 of multivariate regressions.

**Fig. 4:**
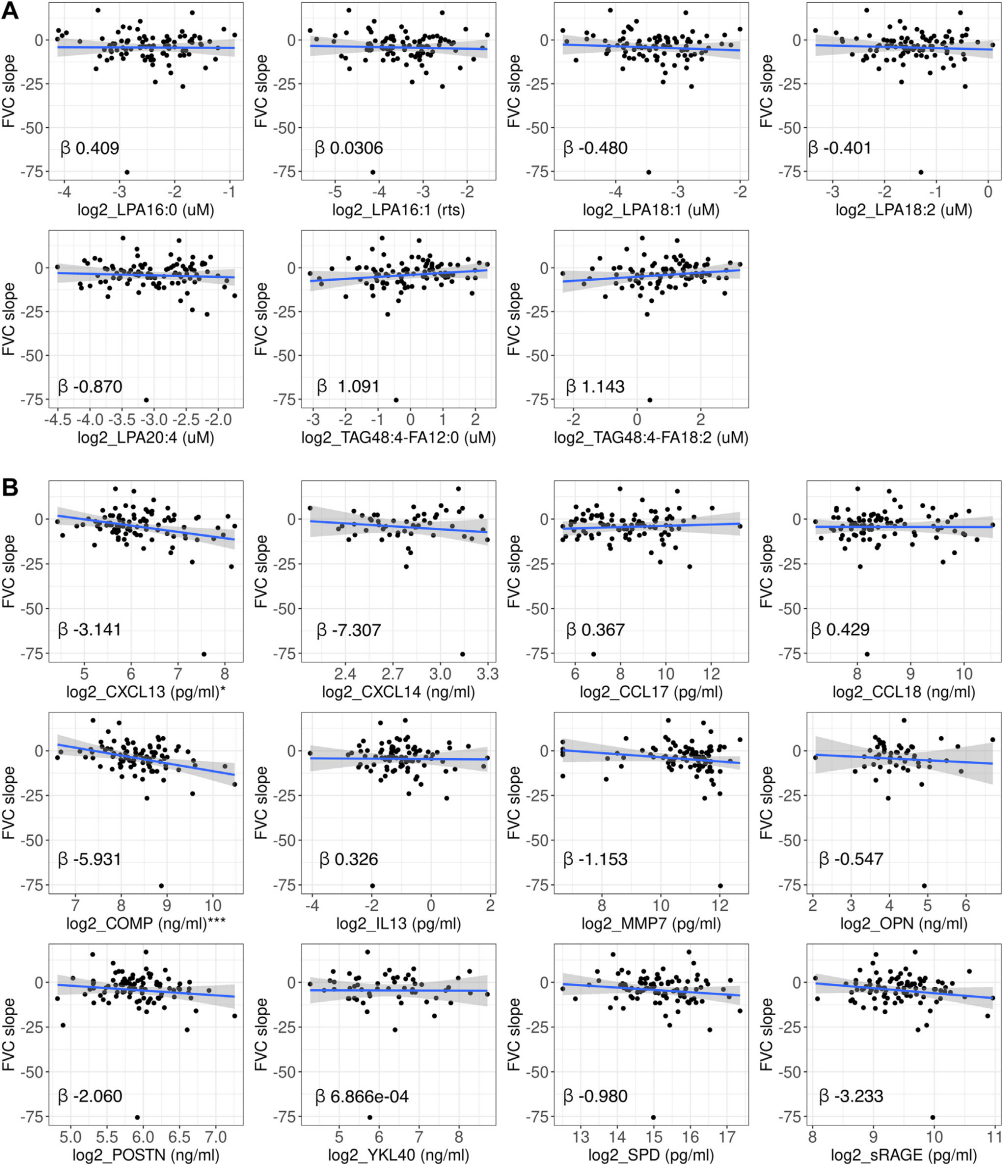
Decline in FVC percentage predicted and baseline biomarker levels. A multivariate linear regression model adjusted for the following covariates: age, sex, baseline FVC %pred, baseline DLCO %pred, and geographical region, was used to assess the association between (A) LPA and TAG and (B) protein biomarkers, with FVC %pred decline calculated as slope, where the y-axis indicated FVC %pred change per year. ∗*P* < 0.05; ∗∗∗*P* < 0.001 of multivariate linear regressions.

FVC %pred decline was not associated with any of the lipid species (Fig. 4A), but with CXCL13 and COMP where patients with higher baseline levels of either of these protein biomarkers had greater FVC %pred declines (*P* < 0.05–<0.001) (Fig. 4B). COMP remained a significant prognostic biomarker (*P* < 0.01) after adjusting for CXCL13 (Table 3).

**Fig. 5:**
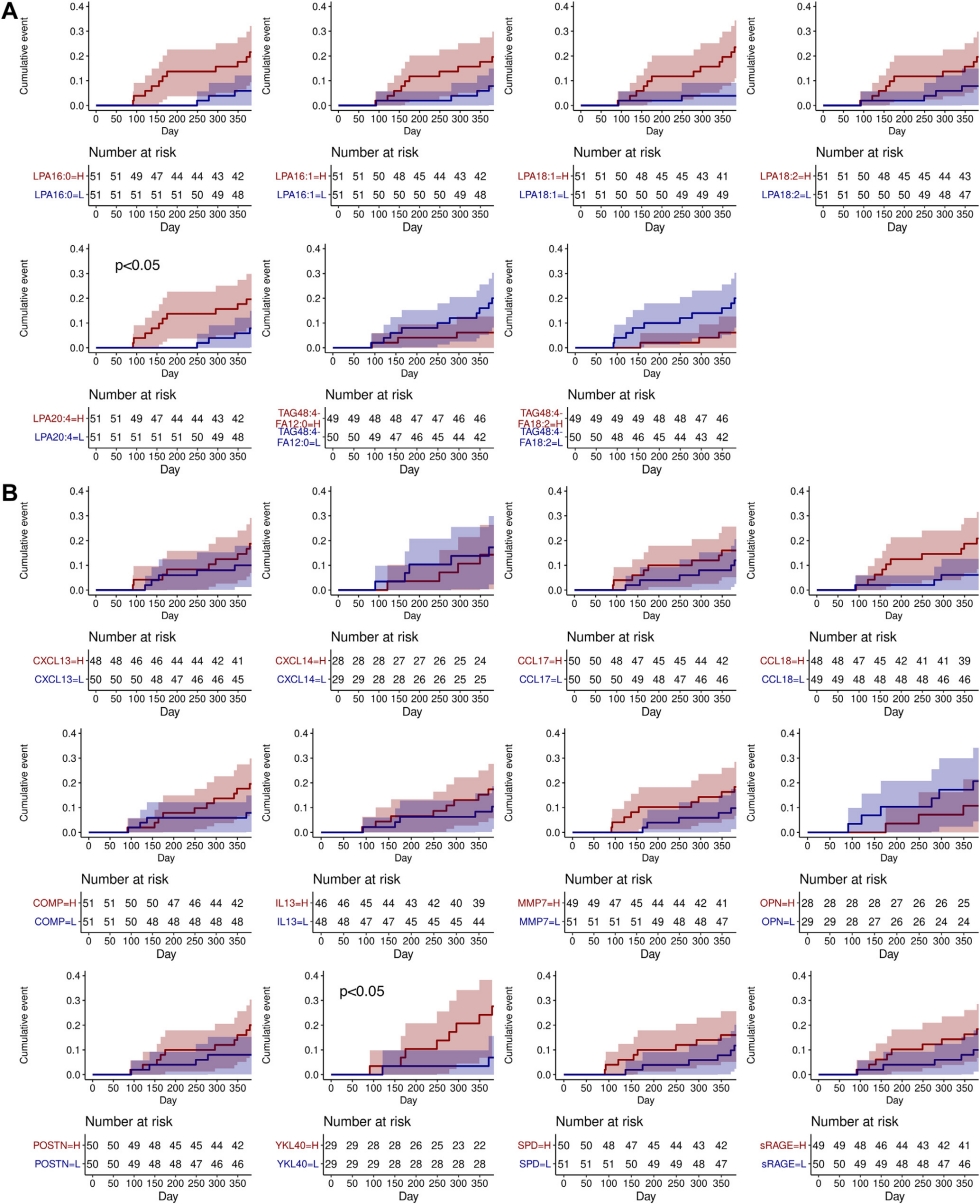
Time to first exacerbation or respiratory hospitalization and baseline (A) lipid or (B) protein biomarker profile. Baseline biomarker profile was fitted to a Cox proportional hazards regression model adjusted for the following covariates: age, sex, baseline FVC %pred, baseline DLCO %pred, and geographical region. Multivariate linear regression *P*-values shown.

Patients with higher levels (≥ median) of LPA20:4 had an earlier time to exacerbation or respiratory hospitalization compared with the biomarker low subgroups (hazard ratio (95% CI)): LPA20:4-high = 5.71 (1.17–27.72) (*P* = 0.031) (Fig. 5A). Among the protein biomarkers, YKL40-high (≥ median) patients had an earlier time to exacerbation or respiratory hospitalization: YKL40-high = 13.61 (1.11–166.18) (*P* = 0.041) (Fig. 5B). In the combined LPA20:4 and YKL40 model, the individual biomarker was no longer significant, albeit YKL40 remained marginally associated (*P* = 0.053) (Table 3).

**Fig. 6:**
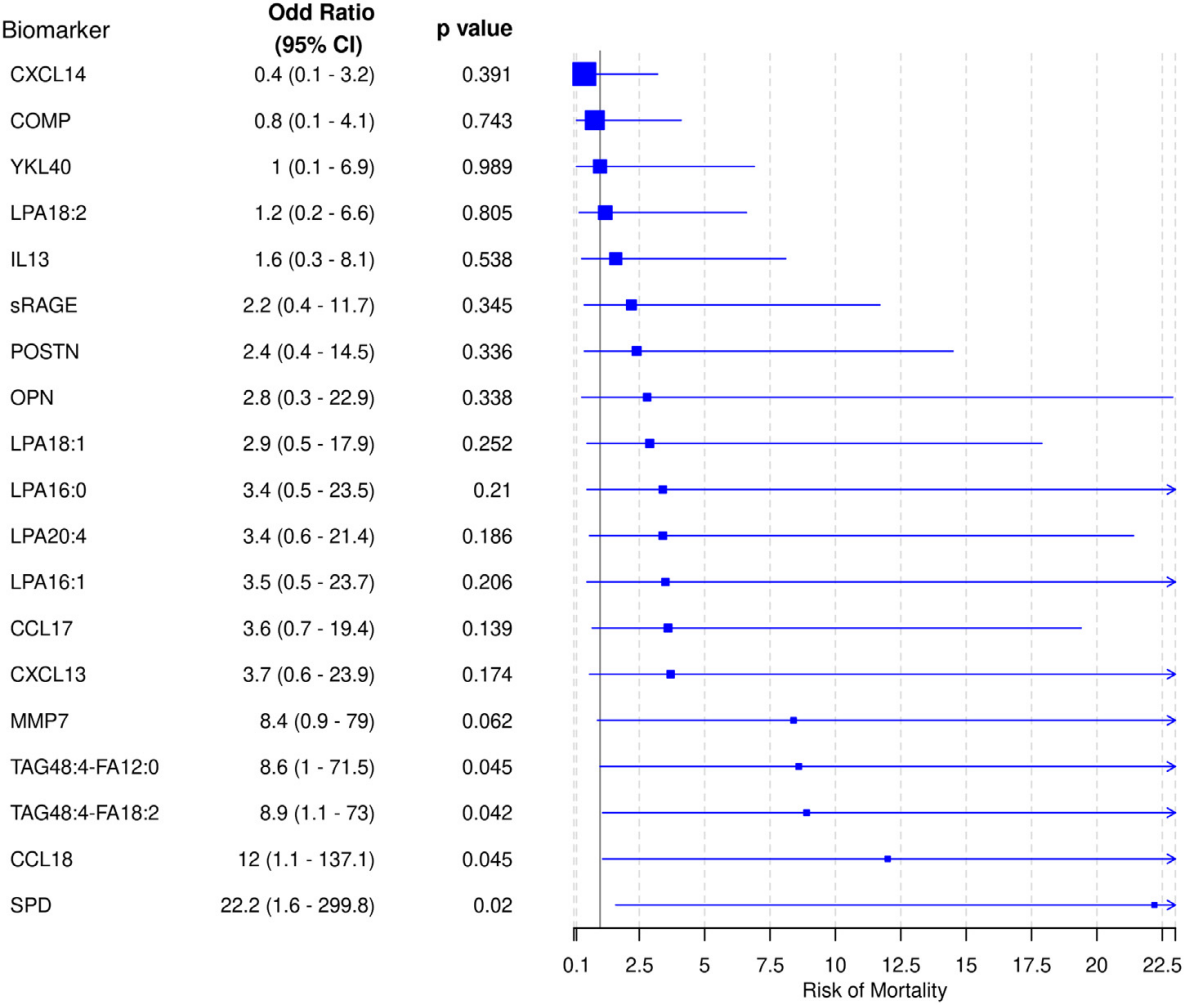
Risk of mortality and baseline biomarker profile. The baseline biomarker profile was fitted to a multivariate logistic regression model adjusted for the following covariates: age, sex, baseline FVC %pred, baseline DLCO %pred, and geographical region. An odds ratio above 1 denotes higher odds of mortality in patients with higher levels (≥median) of biomarker compared to patients with lower levels (<median) of biomarker; specifically for TAG species, an odds ratio above 1 denotes higher odds of mortality in patients with the lower levels (<median) of TAG compared to patients with higher levels of (≥median) TAG. Line arrows denote censored confidence intervals.

The TAG species were prognostic of mortality as patients with lower levels (< median) of the TAG species had increased risk of mortality (odd ratio (95% CI)): TAG48:4-FA12:0-low = 8.6 (1.0–71.5) (*P* = 0.045), TAG48:4-FA18:2-low = 8.9 (1.1–73.0) (*P* = 0.042); conversely, patients with higher levels (≥ median) of SPD or CCL18 had increased mortality risks: SPD-high = 22.2 (1.6–299.8) (*P* = 0.020), CCL18-high = 12.0 (1.1–137.1) (*P* = 0.045) (Fig. 6). CCL18 and SPD remained independent prognostic biomarkers in the combined protein biomarker model; the significant association of CCL18, but not SPD, remained after adjusting for the TAG species (Table 3).

### Prognostic biomarkers of radiographic changes

**Fig. 7:**
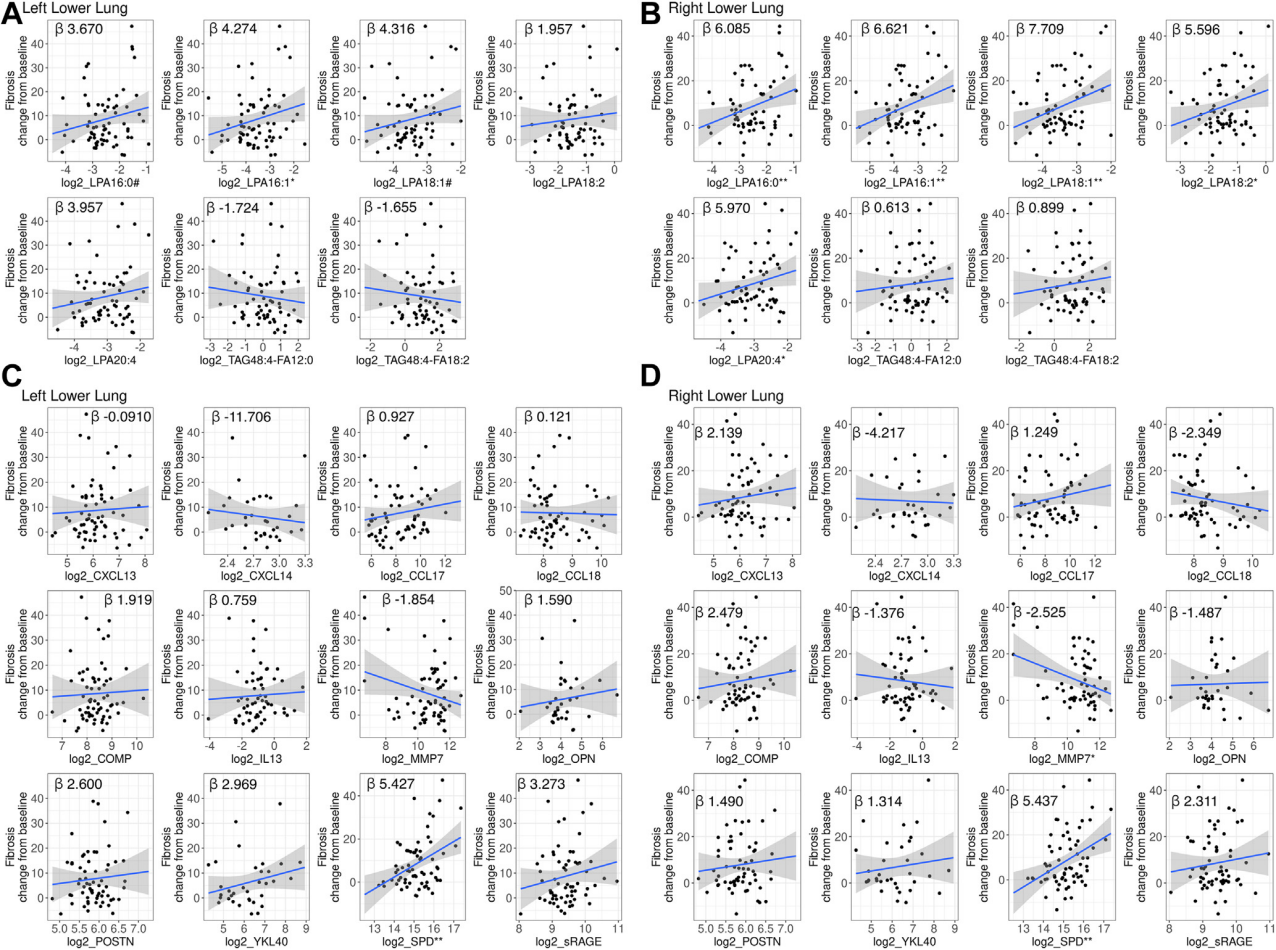
Fibrosis changes in left and right lower lungs by baseline (A and B) lipid and (C and D) protein biomarker profile. A multivariate linear regression model adjusted for the following covariates: age, sex, baseline FVC %pred, baseline DLCO %pred, and geographical region, was used to assess the association between (A and B) lipids, and (C and D) protein biomarkers with fibrosis change from baseline in the (A and C) left lower lungs, or (B and D) right lower lungs. #*P* < 0.1; ∗*P* < 0.05; ∗∗*P* < 0.01 of multivariate linear regressions.

IPF is recognized in HRCT by subpleural lower lobe reticular opacities and honeycombing (21). Honeycombing is considered the end-stage of fibrosis, and its progression is typically fastest in the lower lobes of the lungs (22). Looking into the radiographic changes by lung regions (lower, middle, and upper), there were minimal changes in ground glass (supplemental Fig. S3A) and honeycombing (supplemental Fig. S3B) in the overall patient population by week 72, but increases in fibrosis were observed and occurred mostly in the lower lungs (supplemental Fig. S3C). No association with ground glass or honeycombing changes was observed with the lipid biomarkers (not shown). Interestingly, all of the LPA species were prognostic of fibrosis increase in the lower lungs at week 72, whereas patients with higher baseline levels of LPA had greater increases in fibrosis (*P* < 0.05–*P* < 0.01) (Fig. 7A, B).

The LPA model absolute coefficients ranged from 4.27 to 7.71, which showed that for every twofold increase in baseline LPA levels, fibrosis increases by an additional 4.27%–7.71% in the lower left or right lung in 72 weeks, with all other covariables held constant. TAG species were not prognostic of fibrotic changes. Higher levels of SPD at baseline were also associated with greater increases in fibrosis in the lower lungs (*P* < 0.01) (Fig. 7C, D); surprisingly, higher levels of MMP7 were associated with less fibrotic changes in the right lower lung (*P* < 0.05) (Fig. 7D). In the combined SPD and individual LPA species models, SPD and all the LPA species except LPA20:4, were independent prognostic biomarkers of fibrosis increase in the right lower lung (Table 3). LPA16:1 was the only LPA species prognostic of fibrosis increase in the left lower lung; in the combined SPD and LPA16:1 model, SPD remained an independent prognostic biomarker, LPA16:1 was marginally associated after adjusting for SPD (*P* = 0.0594) (Table 3).

## Discussion

In this study, we identified a number of LPA and TAG species that were significantly dysregulated in patients with IPF. Majority of these lipid species were prognostic of clinical and radiographic outcomes including DLCO decline, exacerbation or respiratory hospitalization, mortality, and fibrosis increase in the lower lungs. Since this study included a subset of patients reported previously (20), a number of reported prognostic protein biomarkers in IPF, that is, CXCL13, POSTN, COMP, YKL40, and SPD, were significantly associated with disease progression which served as benchmarks for the lipid biomarkers. Our study has shown that high LPA species at baseline remained significantly associated with DLCO decline and fibrosis increase following the adjustment for the prognostic protein biomarkers, patient demographics, FVC, and DLCO.

IPF lungs have elevated levels of lipid palmitic acid (C16:0) compared to controls (23). The exogenous addition of palmitic acid to epithelial cells increased reactive oxygen species and apoptosis (24). Interestingly, a deficiency in stearoyl CoA desaturase-1 that catalyzes the conversion of saturated to monounsaturated FA resulted in ER stress and fibrosis in mice (25). These data suggest that lipotoxicity due to the accumulation of saturated FAs may contribute to lung fibrosis by inducing apoptosis in epithelial cells and ER stress. Transcriptomic analyses corroborate the findings as genes involved in lipid metabolism were dysregulated in IPF lungs as well as the alveolar type 2 cells isolated from patients with IPF (26). Alveolar type 2 cells produce pulmonary surfactant which is made up of a complex mixture of lipids (phospholipids, TAG, others) and surfactant proteins; aberration in lipid metabolism in the lungs could be reflected in the respiratory and systemic compartments, as studies have shown that the phospholipid levels in the bronchoalveolar lavage correlate with IPF disease severity (27), and systemic levels of surfactant protein A and D (SPD) are prognostic of mortality in IPF (28). Consistently, SPD was also associated with mortality in this study but the association diminished after adjusting for TAG species. Taken together, the increased levels of LPA and reduced levels of TAG in the systemic circulation observed in this study are likely a reflection of the dysregulated lipid metabolism in IPF lungs.

Systemic LPA could also originate from macrophages or inflammatory cells in the form of vesicles (29, 30) as well as fibroblasts (31). LPA promotes monocyte migration (32) and mediates the differentiation of monocytes to macrophages (33). In this study, a number of the LPA species (LPA16:0, 16:1, 18:1, and 20:4) showed significant associations with inflammation-related biomarkers CCL17 and CCL18. Monocytes preferentially differentiate into M2 macrophages under type II helper T cells (Th2) inflammation, and Th2 chemokines such as CCL17 have been shown to contribute to the development of pulmonary fibrosis in the bleomycin mouse model (34). CCL18 is a biomarker of M2 macrophages with fibrogenic properties and a predictor of FVC decline and mortality (20). The association of LPA species with CCL17 and CCL18 in the systemic circulation suggests that these lipid species might also contribute to disease progression via Th2-mediated responses or M2 macrophage activity leading to fibrosis.

LPA can bind to non-G-protein-coupled receptors such as RAGE to mediate vascular inflammation and platelet activation (35). RAGE is expressed predominantly on alveolar Type 1 cells, endothelial, and vascular smooth muscle cells; soluble RAGE (sRAGE) lacking the transmembrane domain exists as a decoy receptor that binds RAGE ligands to attenuate RAGE-mediated inflammation. The positive correlation between sRAGE and LPA in this study points to a potential feedback loop to dampen inflammation.

YKL40 serum levels and gene expression in the lungs were higher in patients with IPF compared to healthy controls (36); this protein has pluripotent roles ranging from the mediation of endothelial cells migration (37), Th2 inflammation to the enhancement of TGF-β activation (38). LPA has been shown to upregulate YKL40 expression in human osteoblasts in vitro (39), the overlapping signaling pathways between LPA and YKL40 might be the reason that they were not independently prognostic of exacerbation or respiratory hospitalization in this study.

Among the protein biomarkers identified to be prognostic of disease progression by Clynick B *et al.* (40) that were also measured in this study, we observed that only POSTN and SPD were prognostic; POSTN was associated with DLCO decline, while SPD was associated with mortality risk and fibrosis increase in the lower lungs. The negative association between MMP7 and lung fibrosis in this study was unexpected, potentially driven by outliers or patient-specific factors that were not discernible. The discordance between the studies could be due to a number of factors. First, there might be inherent differences in the patient population enrolled in observational studies versus clinical trials, and the biomarkers might reflect different pathobiology at different stages of the disease. Second, in an effort to better understand the connection between molecular pathways and clinical manifestations, we examined each of the clinical measures of progression independently, instead of using the composite measure of FVC, DLCO, and/or death to define a progressive disease (40). Uniquely, our results demonstrated the association of LPA with DLCO decline and radiographic progression that was not observed in any of the protein biomarkers, potentially attributable to the dual roles of LPA in vascular leakage as well as TGF-β activation.

A growing body of evidence implicates the role of increased alveolar-capillary permeability and endothelial dysfunction in IPF pathogenesis (41). Pro-inflammatory and pro-fibrotic mediators including TGF-β1 and LPA activate Rho-kinase signaling in endothelium leading to cytoskeletal reorganization and increased endothelial permeability (41, 42). Inhibiting Rho-kinase signaling by pharmacological inhibitors reduced LPA-induced increases in vascular permeability in mouse models of lung injury (42) and attenuated bleomycin-induced fibrotic response (43). Consistently, the deletion of the LPA1 receptor reduced vascular leakage in a bleomycin mouse model of fibrosis (10). Deterioration in endothelial dysfunction or vascular permeability could potentially manifest clinically as a decline in DLCO, as DLCO is a marker of alveolar-capillary interface integrity that measures the gas transfer capacity of the capillary interface and the volume of blood available for gas exchange (44). The link between DLCO and endothelial dysfunction or vascular permeability may explain why patients with higher levels of LPA had greater declines in DLCO which was accompanied by increases in fibrosis in the lower lungs.

The distribution of fibrosis in IPF lungs has been linked to acute exacerbation and mortality (45, 46). Patients with asymmetrical disease, defined as having fibrosis in one lung that was 1.5-fold greater than that of another lung, had a significantly higher rate of acute exacerbations compared to patients with symmetrical disease (45). Moreover, patients with both lungs affected symmetrically with ground glass during AE-IPF had a higher mortality rate within 6 months (46). The HRCT measurements captured in the study were not amenable to detailed analyses of disease symmetry as reported by Tcherakian C *et al.* (45), but we did observe that HRCT changes were variable by lung zones. Importantly, LPA levels were associated with fibrosis increase in lower lungs, and these changes were accompanied by worse outcomes including an earlier time to AE-IPF or respiratory hospitalization, establishing evidence that the LPA signaling pathway play an important role in these radiographic and clinical manifestations.

The reasons why LPA and TAG species were prognostic of some but not all of the clinical and radiographic outcomes in this study remain unclear. LPA mediates the downstream signaling through receptor activation where six LPA receptors have been characterized that are expressed at different levels on different cell types (9). As a result, both the protective and the pathogenic roles of LPA in the airway have been reported. LPA receptors are required to maintain epithelial barrier function, control allergic lung inflammation (47, 48), and support alveolarization (49). Related to lung pathology, LPA receptor activation leads to lung fibrosis (10, 50), epithelial cell apoptosis (51), inflammatory cytokines production, and neutrophilic infiltration (52). It is unclear if TAG species activate any receptors. Further work is needed to validate the TAG changes using quantitative targeted methods and to investigate the biological processes elicited by the individual LPA and TAG species in the context of IPF.

The study has a number of limitations. First, this was a post hoc analysis of a clinical trial and sampling was incomplete. However, the demographics captured were representative of the overall placebo population. Second, we prioritized lipids with at least a twofold difference compared to healthy for clinical analyses; the differences in some of the clinical outcomes were based on the median level of the LPA and TAG used to subgroup patients, additional work is needed to assess the optimal cutoffs. Finally, the relationship between systemic LPA levels and the lung compartment has not been examined as lung sampling was not collected in this study.

In summary, we identified plasma LPA and TAG species that were significantly dysregulated in IPF patients; these LPA and TAG species were prognostic of clinical outcomes but only LPA species were independent prognostic biomarkers of DLCO decline and fibrosis increase in the lung. Further work is needed to understand the mechanistic processes underlying these observations. IPF has a poor prognosis and patients with AE-IPF reportedly have increased mortality and morbidity. The discovery of these biomarkers may aid in the early identification of high-risk patients with rapid disease progression, which is imperative to prompt the initiation of appropriate treatments to preserve lung function.

## Data availability

The data that support the findings of the study are available from the corresponding author upon reasonable request.

## Supplemental data

This article contains supplemental data.

## Conflict of interest

The authors declare the following financial interests/personal relationships which may be considered as potential competing interests: M. N., Q. L., W. R. W., G. J., W. S., and G. W. T. are employees of Genentech Inc. S. J. Z and J. L. are employees of Roche.
