# Supplementary material for: Bioactive lipid lysophosphatidic acid species are associated with disease progression in idiopathic pulmonary fibrosis
Source: J Lipid Res. 2023 Apr 17;64(6):100375. doi: 10.1016/j.jlr.2023.100375 (PMC10205439; doi:10.1016/j.jlr.2023.100375)
Supplement: Supplemental Tables S1 and S2 and Figures S1 and S2 [file mmc1.docx]

**Bioactive lipid lysophosphatidic acid species are associated with disease progression in idiopathic pulmonary fibrosis**

Margaret Neighbors^a^, Qingling Li^b^, Sha (Joe) Zhu^c^, Jia Liu^c^, Weng Ruh Wong^b^, Guiquan Jia^d^, Wendy Sandoval^b^, Gaik W. Tew^e^

**Supplementary Results**

**Supplemental Table S1. Median cutoffs used to subgroup IPF patients into biomarker high (≥ median) and low (< median) subgroups**.

| Lipid species | Male | Female |
| --- | --- | --- |
| LPA16:0 (uM) | 0.173 | 0.227 |
| LPA16:1 (rts) | 0.078 | 0.121 |
| LPA18:1 (uM) | 0.098 | 0.102 |
| LPA18:2 (uM) | 0.359 | 0.408 |
| LPA20:4 (uM) | 0.130 | 0.120 |
| TG48:4-FA12:0 (uM) | 1.186 | 0.820 |
| TG48:4-FA18:2 (uM) | 2.173 | 1.607 |

uM=microMolar; rts=ratio-to-standard.

**Supplemental Table S2. Differential expression of lipid species between IPF and healthy controls**

| Upregulated in IPF | Fold Change IPF/control | log_10_FDR |
| --- | --- | --- |
| CE(20:0) | 1.999 | 1.363 |
| CE(20:1) | 1.472 | 1.899 |
| CE(22:2) | 1.881 | 2.265 |
| CE(22:6) | 1.190 | 1.512 |
| DAG(12:0/18:1) | 1.781 | 17.818 |
| LPC(14:0) | 1.486 | 3.194 |
| LPC(15:0) | 1.459 | 4.594 |
| LPC(16:0) | 1.620 | 7.718 |
| LPC(16:1) | 1.977 | 8.807 |
| LPC(17:0) | 1.612 | 7.818 |
| LPC(18:0) | 1.703 | 8.430 |
| LPC(18:1) | 1.594 | 6.549 |
| LPC(20:0) | 1.296 | 2.893 |
| LPC(20:1) | 1.679 | 6.453 |
| LPC(20:2) | 1.427 | 4.525 |
| LPC(20:3) | 1.262 | 2.200 |
| LPC(20:4) | 1.270 | 2.283 |
| LPC(20:5) | 1.594 | 1.796 |
| LPC(22:4) | 1.304 | 2.477 |
| LPC(22:5) | 1.325 | 3.143 |
| LPC(22:6) | 1.534 | 4.671 |
| LPE(14:0) | 1.158 | 1.573 |
| LPE(15:0) | 1.194 | 1.995 |
| LPE(16:0) | 1.319 | 3.001 |
| LPE(16:1) | 1.299 | 2.377 |
| LPE(17:0) | 1.341 | 4.525 |
| LPE(18:0) | 1.630 | 4.224 |
| LPE(20:0) | 1.302 | 3.070 |
| LPE(20:1) | 1.415 | 3.575 |
| LPE(20:2) | 1.255 | 2.418 |
| LPE(22:6) | 1.248 | 2.880 |
| PC(12:0/16:1) | 1.244 | 2.463 |
| PC(12:0/18:1) | 1.365 | 3.027 |
| PC(12:0/18:3) | 1.308 | 1.637 |
| PC(12:0/18:4) | 1.164 | 1.420 |
| PC(12:0/20:1) | 1.273 | 2.814 |
| PC(12:0/20:2) | 1.192 | 1.629 |
| PC(12:0/20:3) | 1.276 | 2.714 |
| PC(12:0/20:4) | 1.274 | 2.648 |
| PC(12:0/20:5) | 1.340 | 3.726 |
| PC(12:0/22:2) | 1.202 | 1.774 |
| PC(12:0/22:4) | 1.177 | 1.363 |
| PC(12:0/22:5) | 1.287 | 2.965 |
| PC(12:0/22:6) | 1.175 | 1.509 |
| PC(14:0/18:4) | 1.195 | 1.643 |
| PC(14:0/20:1) | 1.220 | 2.194 |
| PC(14:0/20:5) | 1.282 | 1.371 |
| PC(14:0/22:1) | 1.182 | 1.796 |
| PC(14:0/22:2) | 1.188 | 1.611 |
| PC(14:0/22:6) | 1.236 | 1.421 |
| PC(15:0/14:1) | 1.238 | 2.557 |
| PC(15:0/20:5) | 1.246 | 1.729 |
| PC(15:0/22:4) | 1.199 | 2.071 |
| PC(16:0/22:1) | 1.299 | 2.531 |
| PC(16:0/22:2) | 1.404 | 2.351 |
| PC(17:0/14:1) | 1.246 | 2.860 |
| PC(17:0/16:1) | 1.171 | 1.486 |
| PC(17:0/18:3) | 1.237 | 2.430 |
| PC(17:0/22:4) | 1.187 | 2.112 |
| PC(18:0/14:1) | 1.211 | 2.199 |
| PC(18:0/18:1) | 1.183 | 1.455 |
| PC(18:0/20:0) | 1.375 | 3.863 |
| PC(18:0/20:1) | 1.184 | 2.327 |
| PC(18:0/22:1) | 1.296 | 3.584 |
| PC(18:0/22:2) | 1.406 | 4.592 |
| PC(18:0/22:6) | 1.332 | 2.091 |
| PC(18:1/14:1) | 1.234 | 2.597 |
| PC(18:1/16:1) | 1.334 | 2.207 |
| PC(18:1/18:1) | 1.355 | 2.454 |
| PC(18:1/18:4) | 1.216 | 2.283 |
| PC(18:1/20:1) | 1.233 | 2.131 |
| PC(18:1/20:2) | 1.392 | 3.485 |
| PC(18:1/20:3) | 1.529 | 4.677 |
| PC(18:1/20:4) | 1.360 | 2.430 |
| PC(18:1/20:5) | 1.562 | 1.586 |
| PC(18:1/22:1) | 1.255 | 2.841 |
| PC(18:1/22:2) | 1.257 | 2.888 |
| PC(18:1/22:4) | 1.592 | 4.204 |
| PC(18:1/22:5) | 1.659 | 4.711 |
| PC(18:1/22:6) | 1.668 | 5.123 |
| PC(18:2/14:1) | 1.337 | 3.863 |
| PC(18:2/18:4) | 1.256 | 3.194 |
| PC(18:2/22:1) | 1.234 | 2.228 |
| PC(18:2/22:2) | 1.186 | 1.693 |
| PC(18:2/22:6) | 1.192 | 1.799 |
| PC(20:0/16:1) | 1.264 | 1.825 |
| PC(20:0/18:1) | 1.218 | 1.408 |
| PC(20:0/18:4) | 1.211 | 1.973 |
| PC(20:0/20:2) | 1.217 | 2.022 |
| PC(20:0/22:2) | 1.228 | 2.074 |
| PC(20:0/22:4) | 1.260 | 2.329 |
| PC(20:0/22:5) | 1.189 | 1.380 |
| PC(20:0/22:6) | 1.292 | 2.531 |
| PE(14:0/14:1) | 1.248 | 3.027 |
| PE(14:0/16:1) | 1.247 | 2.880 |
| PE(14:0/18:3) | 1.215 | 2.542 |
| PE(14:0/18:4) | 1.203 | 2.569 |
| PE(14:0/20:2) | 1.172 | 1.796 |
| PE(14:0/20:3) | 1.192 | 2.204 |
| PE(14:0/20:4) | 1.173 | 1.729 |
| PE(14:0/20:5) | 1.183 | 2.043 |
| PE(14:0/22:1) | 1.185 | 1.903 |
| PE(14:0/22:2) | 1.161 | 1.555 |
| PE(14:0/22:4) | 1.217 | 2.719 |
| PE(15:0/14:1) | 1.178 | 2.267 |
| PE(15:0/16:1) | 1.177 | 2.199 |
| PE(15:0/18:1) | 1.230 | 2.864 |
| PE(15:0/18:2) | 1.168 | 1.633 |
| PE(15:0/18:3) | 1.196 | 2.384 |
| PE(15:0/18:4) | 1.246 | 3.118 |
| PE(15:0/20:3) | 1.191 | 2.240 |
| PE(15:0/20:4) | 1.140 | 1.490 |
| PE(15:0/22:1) | 1.269 | 3.306 |
| PE(15:0/22:2) | 1.172 | 2.181 |
| PE(15:0/22:4) | 1.179 | 2.233 |
| PE(15:0/22:5) | 1.215 | 2.542 |
| PE(15:0/22:6) | 1.237 | 3.104 |
| PE(16:0/14:1) | 1.193 | 2.055 |
| PE(16:0/15:0) | 1.176 | 1.796 |
| PE(16:0/17:0) | 1.177 | 2.061 |
| PE(16:0/18:4) | 1.289 | 4.224 |
| PE(16:0/20:1) | 1.198 | 1.934 |
| PE(16:0/22:1) | 1.214 | 2.719 |
| PE(16:0/22:2) | 1.228 | 2.999 |
| PE(17:0/14:1) | 1.218 | 2.674 |
| PE(17:0/16:1) | 1.240 | 2.723 |
| PE(17:0/18:3) | 1.196 | 2.112 |
| PE(17:0/18:4) | 1.275 | 4.069 |
| PE(17:0/20:3) | 1.208 | 2.402 |
| PE(17:0/20:5) | 1.223 | 2.458 |
| PE(17:0/22:2) | 1.185 | 2.244 |
| PE(17:0/22:5) | 1.223 | 2.377 |
| PE(17:0/22:6) | 1.204 | 1.960 |
| PE(18:0/14:0) | 1.216 | 1.832 |
| PE(18:0/15:0) | 1.168 | 1.612 |
| PE(18:0/17:0) | 1.193 | 1.948 |
| PE(18:0/18:4) | 1.272 | 3.541 |
| PE(18:0/22:2) | 1.313 | 3.428 |
| PE(18:1/14:1) | 1.212 | 1.612 |
| PE(18:1/16:1) | 1.249 | 1.934 |
| PE(18:1/18:4) | 1.215 | 2.569 |
| PE(18:1/20:1) | 1.296 | 2.569 |
| PE(18:1/20:2) | 1.265 | 2.287 |
| PE(18:1/20:3) | 1.329 | 1.611 |
| PE(18:1/22:0) | 1.498 | 3.194 |
| PE(18:1/22:1) | 1.260 | 3.575 |
| PE(18:1/22:2) | 1.315 | 4.934 |
| PE(18:1/22:4) | 1.555 | 2.454 |
| PE(18:1/22:5) | 1.330 | 1.805 |
| PE(18:2/18:4) | 1.209 | 2.484 |
| PE(18:2/20:3) | 1.230 | 2.569 |
| PE(18:2/20:5) | 1.218 | 2.719 |
| PE(18:2/22:4) | 1.290 | 2.879 |
| PE(18:2/22:5) | 1.236 | 2.942 |
| PE(18:2/22:6) | 1.319 | 4.657 |
| PE(O-16:0/14:1) | 1.168 | 1.649 |
| PE(O-16:0/16:1) | 1.238 | 2.557 |
| PE(O-16:0/18:0) | 1.205 | 1.894 |
| PE(O-16:0/18:4) | 1.188 | 2.108 |
| PE(O-16:0/20:1) | 1.179 | 2.181 |
| PE(O-16:0/20:2) | 1.168 | 1.713 |
| PE(O-16:0/22:2) | 1.162 | 1.934 |
| PE(O-18:0/14:1) | 1.163 | 1.647 |
| PE(O-18:0/16:1) | 1.165 | 1.469 |
| PE(O-18:0/18:4) | 1.167 | 1.796 |
| PE(O-18:0/20:1) | 1.181 | 2.233 |
| PE(O-18:0/20:2) | 1.186 | 2.183 |
| PE(O-18:0/22:2) | 1.185 | 1.970 |
| PE(P-14:0/18:0) | 1.193 | 1.823 |
| PE(P-14:0/18:1) | 1.257 | 3.436 |
| PE(P-14:1/18:1) | 1.198 | 2.216 |
| PE(P-16:0/14:1) | 1.185 | 2.007 |
| PE(P-16:0/18:0) | 1.181 | 2.254 |
| PE(P-16:0/18:4) | 1.180 | 2.291 |
| PE(P-16:0/20:1) | 1.150 | 1.408 |
| PE(P-16:0/20:2) | 1.225 | 2.244 |
| PE(P-16:0/22:1) | 1.199 | 2.214 |
| PE(P-16:0/22:2) | 1.222 | 2.880 |
| PE(P-16:1/18:1) | 1.331 | 3.826 |
| PE(P-18:0/14:1) | 1.225 | 2.493 |
| PE(P-18:0/16:1) | 1.299 | 1.733 |
| PE(P-18:0/18:0) | 1.225 | 2.999 |
| PE(P-18:0/18:4) | 1.290 | 4.224 |
| PE(P-18:0/20:1) | 1.258 | 3.436 |
| PE(P-18:0/20:2) | 1.258 | 2.508 |
| PE(P-18:0/22:2) | 1.283 | 3.575 |
| PE(P-18:1/14:1) | 1.142 | 1.406 |
| PE(P-18:1/16:1) | 1.422 | 3.265 |
| PE(P-18:1/18:0) | 1.191 | 2.183 |
| PE(P-18:1/18:4) | 1.151 | 1.406 |
| PE(P-18:1/20:1) | 1.247 | 3.381 |
| PE(P-18:1/20:2) | 1.313 | 4.308 |
| PE(P-18:1/20:3) | 1.256 | 1.367 |
| PE(P-18:1/22:1) | 1.202 | 2.204 |
| PE(P-18:1/22:2) | 1.134 | 1.380 |
| PE(P-18:1/22:4) | 1.725 | 2.719 |
| PE(P-18:1/22:5) | 1.250 | 1.782 |
| PE(P-18:1/22:6) | 1.291 | 1.808 |
| PE(P-18:2/22:6) | 1.193 | 1.896 |
| SM(18:1) | 1.237 | 2.942 |
| TAG52:3-FA16:1 | 1.407 | 1.358 |
| LPA16:0(uM) | 4.256 | 11.292 |
| LPA18:1(uM) | 2.185 | 7.778 |
| LPA18:2(uM) | 3.380 | 10.028 |
| LPA20:4(uM) | 2.686 | 9.767 |
| LPA 16:1 (rts) | 5.801 | 9.524 |
| Downregulated in IPF | Fold change IPF/control | log_10_FDR |
| DAG(18:1/18:2) | 0.886 | 1.406 |
| DAG(18:2/18:3) | 0.900 | 2.295 |
| DAG(18:2/20:4) | 0.842 | 2.402 |
| PC(18:2/18:3) | 0.832 | 1.400 |
| PE(18:0/18:2) | 0.876 | 1.755 |
| PE(O-16:0/22:5) | 0.812 | 2.040 |
| TAG44:2-FA18:2 | 0.583 | 1.782 |
| TAG46:2-FA18:2 | 0.570 | 1.658 |
| TAG46:3-FA18:2 | 0.651 | 1.510 |
| TAG46:4-FA18:2 | 0.537 | 2.254 |
| TAG48:3-FA12:0 | 0.560 | 1.888 |
| TAG48:3-FA18:1 | 0.638 | 1.375 |
| TAG48:3-FA18:2 | 0.705 | 1.305 |
| TAG48:4-FA12:0 | 0.457 | 2.301 |
| TAG48:4-FA18:2 | 0.451 | 2.274 |
| TAG48:5-FA18:3 | 0.622 | 1.414 |
| TAG50:4-FA14:0 | 0.699 | 1.607 |
| TAG50:5-FA14:0 | 0.693 | 1.510 |
| TAG54:7-FA18:2 | 0.587 | 1.372 |
| TAG54:7-FA18:3 | 0.593 | 1.608 |
| TAG54:8-FA18:3 | 0.631 | 1.406 |
| TAG56:8-FA20:4 | 0.764 | 1.304 |

FDR=false discovery rate of multivariate regression adjusted for age and sex.


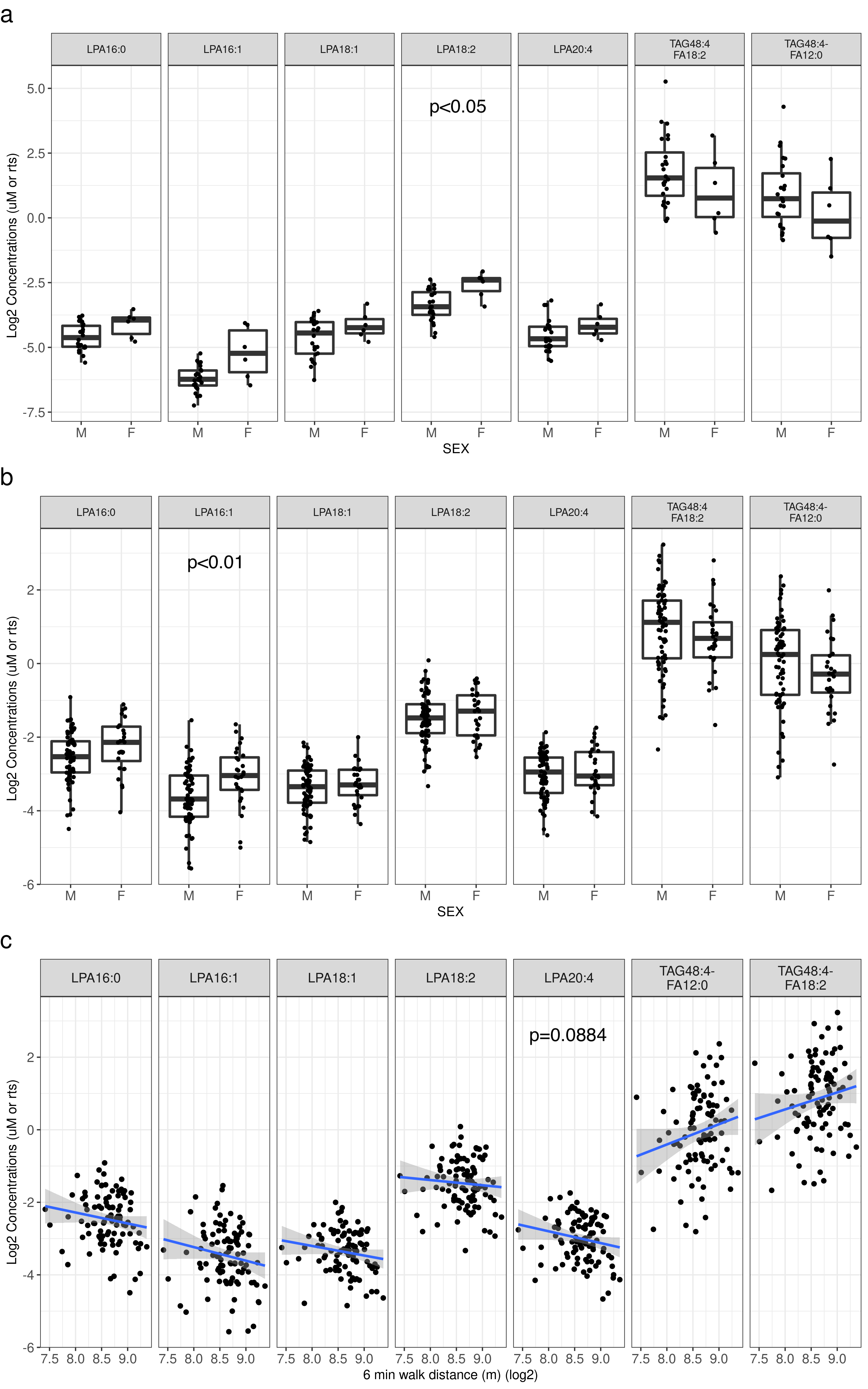


**Supplemental Figure S1. Baseline LPA and TAG characteristics.** Baseline lipid levels by sex in (A) healthy controls, and (B) IPF patients. Student’s t-test p-values shown. (C) Multivariate linear regression adjusted for age, sex and geographic region was used to assess the association of lipids with baseline 6-minute walk distance (meter). F=females; M=males; rts=ratio-to-standard; uM=micromolar.

**
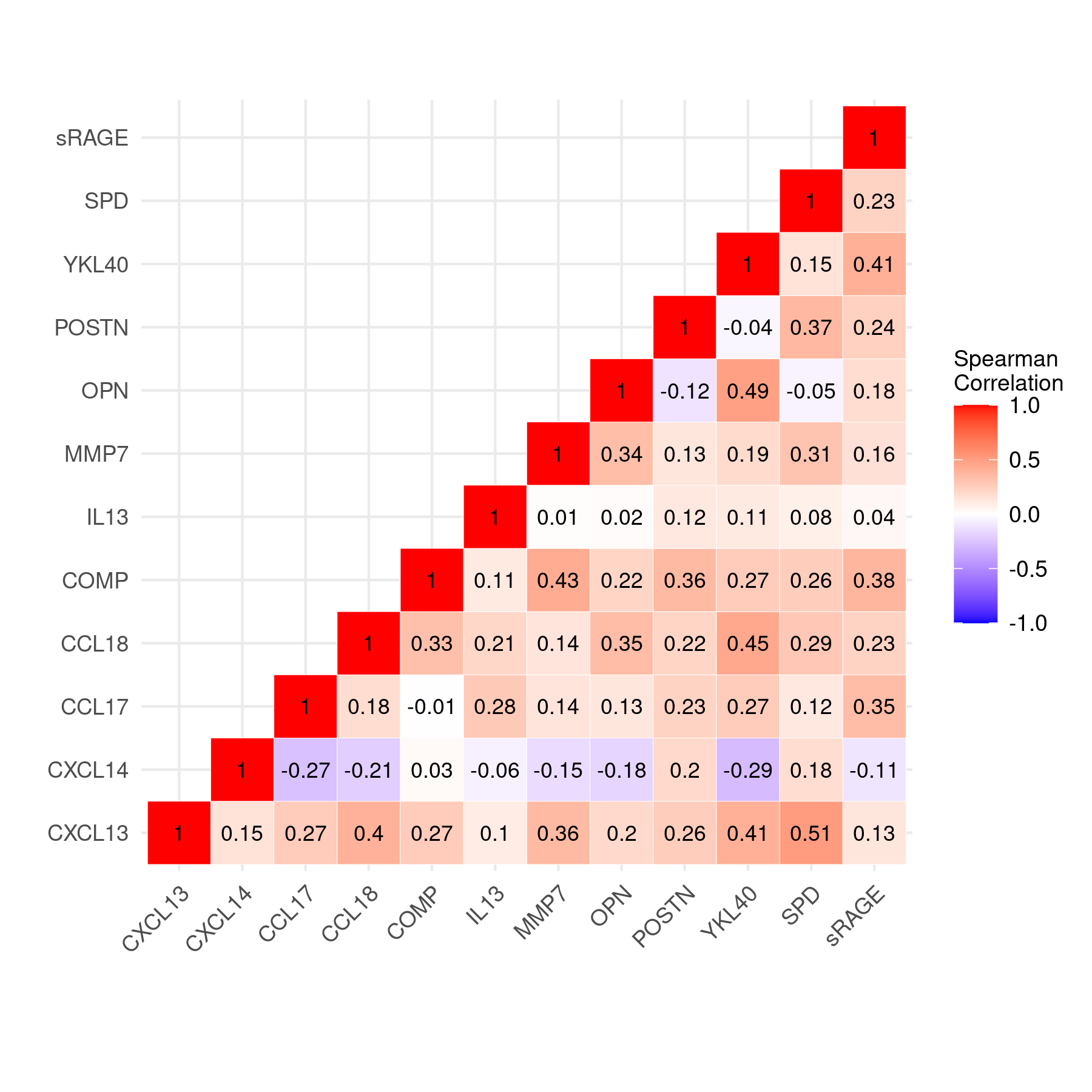
**

**Supplemental Figure S2. Baseline correlation of protein biomarkers.** Spearman’s rho value shown.


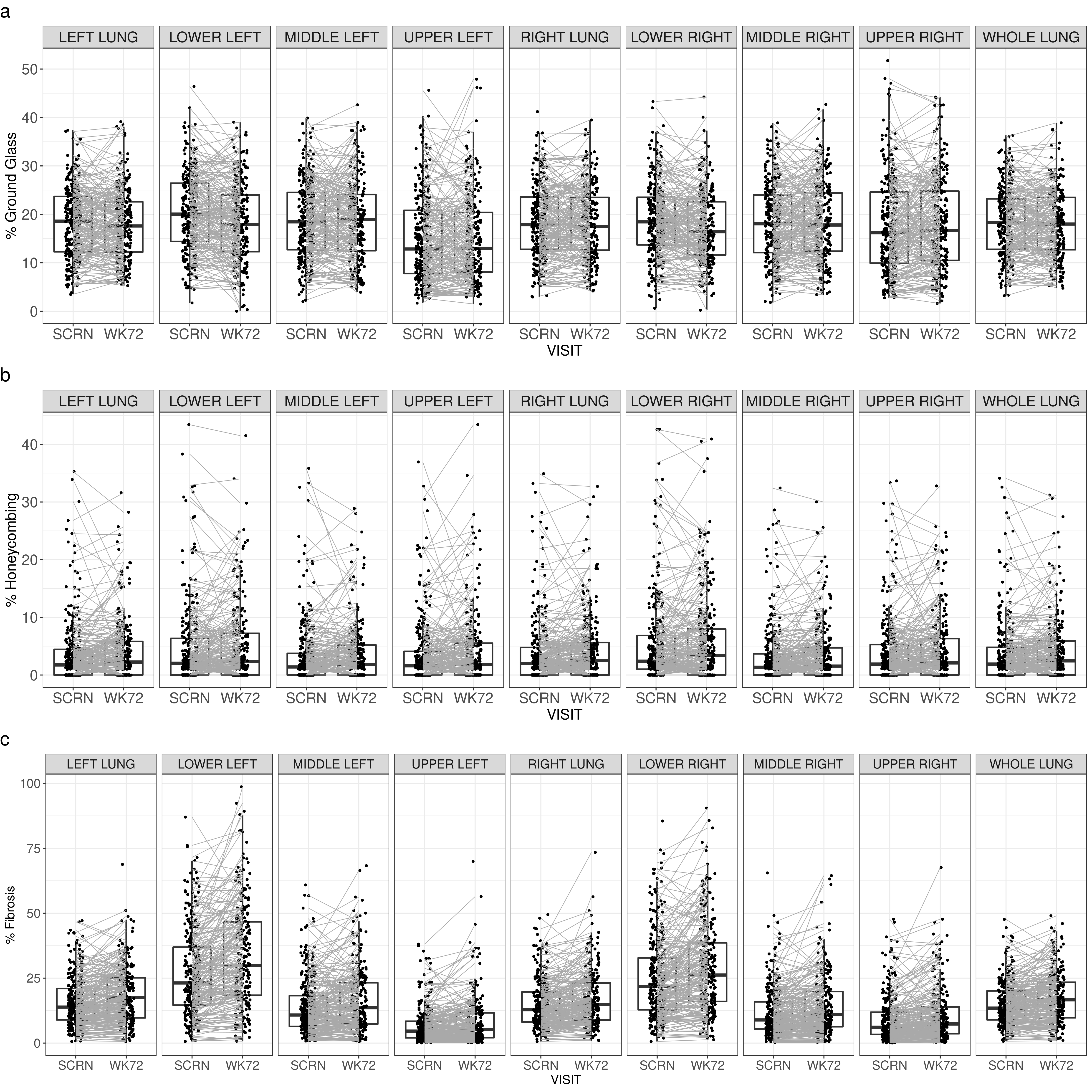


**Supplemental Figure S3. Radiographic changes and lung regions.** The proportion of (a) ground glass opacity, (b) honeycombing, and (c) fibrosis in the different regions of the lungs at screen visit (SCRN) and week 72 (WK72) were shown. Median and interquartile ranges of these radiographic metrics were shown as boxplot, with grey lines connecting the individual patients.
